# The integrated effect of moderate exercise on coronary heart disease

**DOI:** 10.5830/CVJA-2016-058

**Published:** 2017

**Authors:** Marc J Mathews,, Edward H Mathews, George E Mathews

**Affiliations:** Centre for Research and Continued Engineering Development, North-West University, Potchefstroom, South Africa; Centre for Research and Continued Engineering Development, North-West University, Potchefstroom, South Africa; Centre for Research and Continued Engineering Development, North-West University, Potchefstroom, South Africa

**Keywords:** moderate exercise, biomarkers, integrated model

## Abstract

**Background:**

Moderate exercise is associated with a lower risk for coronary heart disease (CHD). A suitable integrated model of the CHD pathogenetic pathways relevant to moderate exercise may help to elucidate this association. Such a model is currently not available in the literature.

**Methods:**

An integrated model of CHD was developed and used to investigate pathogenetic pathways of importance between exercise and CHD. Using biomarker relative-risk data, the pathogenetic effects are representable as measurable effects based on changes in biomarkers.

**Results:**

The integrated model provides insight into higherorder interactions underlying the associations between CHD and moderate exercise. A novel ‘connection graph’ was developed, which simplifies these interactions. It quantitatively illustrates the relationship between moderate exercise and various serological biomarkers of CHD. The connection graph of moderate exercise elucidates all the possible integrated actions through which risk reduction may occur.

**Conclusion:**

An integrated model of CHD provides a summary of the effects of moderate exercise on CHD. It also shows the importance of each CHD pathway that moderate exercise influences. The CHD risk-reducing effects of exercise appear to be primarily driven by decreased inflammation and altered metabolism.

## Background

Coronary heart disease (CHD) is known to be the major cause of death globally.^1^ However, it is well documented that regular moderate physical exercise is associated with fewer CHD events in symptomatic^2^ and asymptomatic^3^,^4^ subjects. The precise mechanisms underlying this inverse association are unclear. However, it is apparent that CHD risk may be substantially mediated, through moderate exercise, by changes in blood pressure, insulin resistance and glucose intolerance, systemic inflammation, triglyceride concentrations, low highdensity lipoprotein (HDL) levels and obesity.^4^,^5^

It may therefore prove beneficial to quantify and elucidate the underlying pathogenetic effect of moderate exercise on the pathogenesis of CHD. Using a previously described integrated model of CHD,^6^,^7^ we investigated the interconnectivity of moderate exercise and the pathogenesis and pathophysiological attributed to CHD.

## Methods

An integrated model was developed as part of a larger research project.^6^ This project has partially been described in previous articles dealing with certain subsets of the research.^7^-^9^ Briefly, a systematic review of the literature post-1998 and including highly cited articles was conducted for CHD pathogenesis, health factors, biomarkers and pharmacotherapeutics. This research was combined to develop the integrated model of CHD.

During the systematic literature review, PubMed, Science Direct, Ebsco Host and Google Scholar were searched for publications with ‘coronary heart disease’ or ‘coronary artery disease’ or ‘cardiovascular disease’ or ‘CHD’ as a keyword and combinations with ‘lifestyle effects’, ‘relative risk prediction’, ‘network analysis’, ‘pathway analysis’, ‘interconnections’, ‘systems biology’, ‘pathogenesis’, ‘biomarkers’, ‘conventional biomarkers’, ‘drugs’, ‘therapeutics’, ‘pharmacotherapeutics’, ‘hypercoagulability’, ‘hypercholesterolaemia’, ‘hyperglycaemia’, ‘hyperinsulinaemia’, ‘inflammation’ and ‘hypertension’ in the title of the study.

Also searched were all major relevant speciality journals in the areas of cardiology, alcohol consumption, nutrition, cigarette smoking, physical exercise, oral health, psychological stress, depression, sleep disorders, endocrinology, psychoneuroendocrinology, systems biology, physiology, periodontology, CHD, the metabolic syndrome and diabetes.

The health factors in the integrated model were considered as lifestyle effects or co-morbid health disorders that have been associated with statistically significant increases or decreases in CHD risk. This resulted in nine health factors being considered in the model, namely alcohol, food, exercise, smoking, oral health, stress, depression, insomnia and sleep apnoea.

The biomarkers considered for the integrated model were mainly those whose measurement has been associated with statistically significant increases or decreases in CHD risk. This resulted in 23 biomarkers being considered in the model, namely triglycerides, low-density lipoprotein (LDL), HDL, apolipoprotein-B (Apo B), leptin, high-sensitivity C-reactive protein (hsCRP), interleukin-6 (IL-6), tumour necrosis factor-α (TNF-α), growth-differentiation factor-15 (GDF- 15), osteoprotegerin (OPG), myeloperoxidase (MPO), B-type natriuretic peptide (BNP), homocysteine, fibrinogen, troponins, urinary albumin-to-creatinine ratio (ACR), glycosylated haemoglobin (_HbA1c_), insulin-like growth factor-1 (IGF-1), adiponectin, cortisol, brain-derived neurotrophic factor (BDNF) and insulin resistance.

In brief, the systematic review of the literature revealed the pathological effects of various health factors on the pathogenesis of CHD. This information was combined to form a visual representation of the pathogenesis of CHD as it is affected by these health factors. The biomarkers were included in the visual representation to show functionally measurable aspects of the pathogenesis.[Bibr R06],^7^ This visual representation presents an integrated model of CHD.

This integrated model of CHD schematically illustrates the complexity of CHD and shows all theoretical pathogenetic pathways between health factors and CHD. The model has been previously used to describe the effects of high-carbohydrate diets on CHD,^7^ and the possible mechanisms through which antidepressants^9^ and moderate alcohol consumption8 may reduce CHD risk.

In this study the integrated model was used to describe the integrated effects of exercise on the pathogenesis of CHD. Furthermore, the effect of exercise on CHD was investigated by analysing the effect that exercise has been shown to have on measurable and quantifiable biomarkers.

## Statistical analysis

It must be noted that some of the relative risk (RR) values in this article differ from convention. The need for this comes as a result of the visual scaling of the traditional RR. Traditionally, if one plots an RR = 3 and RR = 0.33, respectively, one does not ‘look’ three times worse and the other three times better than the normal RR = 1. The reason is that the scales for the positive and negative effects are not numerically similar. A graph of ‘good’ and ‘bad’ RR can therefore be deceptive for the untrained person, for example a patient.

This article rather uses the method that the conventional RR = 3 is three times worse than the normal RR = 1, while the conventional RR = 0.33 means that the patient’s position is three times better than the normal RR = 1. Therefore, in summary, a conventional RR = 3 is presented as per normal, as a three-fold increase in risk and a conventional RR = 0.33 is presented as a three-fold decrease in risk (1/0.33 = 3).

## Results

## Integrated model of coronary heart disease

**Fig. 1.**
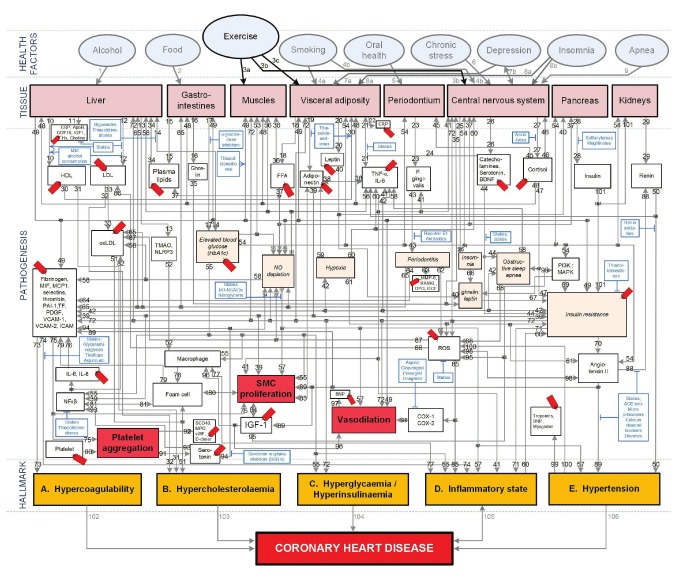
Conceptual model of general health factors, salient CHD pathogenetic pathways and CHD hallmarks. (From: M Mathews, L Liebenberg, E Mathews. How do high glycemic load diets influence coronary heart disease? Nutr Metab 2015; 12(1): 6.7) The affective pathway of pharmacotherapeutics (blue boxes) is shown in Fig. 1, and salient serological biomarkers are indicated by the tags ( ). The blunted arrows denote antagonise or inhibit, and pointed arrows denote up-regulate or facilitate. ACE, angiotensin converting enzyme; BDNF, brain-derived neurotrophic factor; β-blocker, beta-adrenergic antagonists; BNP, B-type natriuretic peptide; COX, cyclooxygenase; CRP, C-reactive protein; D-dimer, fibrin degradation product D; FFA, free fatty acids; GCF, gingival crevicular fluid; HbA1c, glycosylated haemoglobin A1c; HDL, high-density lipoprotein; Hs, homocysteine; ICAM, intracellular adhesion molecule; IGF-1, insulin-like growth factor-1; IL, interleukin; LDL, low-density lipoprotein; MAPK, mitogen-activated protein (MAP) kinase; MCP, monocyte chemo-attractant protein; MIF, macrophage migration inhibitory factor; MMP, matrix metalloproteinase; MPO, myeloperoxidase; NFκβ, nuclear factor-κβ; NLRP3, Inflammasome responsible for activation of inflammatory processes as well as epithelial cell regeneration and microflora; NO, nitric oxide; NO-NSAIDs, combinational NO-non-steroidal anti-inflammatory drug; OPG, osteoprotegerin; oxLDL, oxidised LDL; PAI, plasminogen activator inhibitor; PDGF, platelet-derived growth factor; P gingivalis, Porphyromonas gingivalis; PI3K, phosphatidylinositol 3-kinase; RANKL, receptor activator of nuclear factor kappa-beta ligand; ROS, reactive oxygen species; SCD-40, recombinant human sCD40 ligand; SMC, smooth muscle cell; SSRI, serotonin reuptake inhibitors; TF, tissue factor; TMAO, an oxidation product of trimethylamine (TMA); TNF-α , tumour necrosis factor-α; VCAM, vascular cell adhesion molecule; vWF, von Willebrand factor.

The integrated model of CHD that was developed in previous studies is presented in Fig. 1. The pathways (pathogenesis of CHD) within the integrated model can be tracked from where a chosen health factor influences the relevant tissue, to the end state of CHD. The pathways are therefore a visual representation of previously published knowledge. Salient serological biomarkers (shown in Fig. 1 as ) and pharmacotherapeutics (shown in Fig. 1 as ) that act on the pathways are further indicated in Fig. 1.

The focus of this review is on using the integrated model to describe the interconnections of moderate exercise on the pathogenesis of CHD. Therefore a more detailed discussion of Fig. 1, relevant to exercise, is given in the next section. This review therefore attempts to quantify the CHD effect of moderate exercise by the connection of these to an array of biomarkers that represent increasing or decreasing CHD risk.

## Pathogenetic effects of physical exercise

In order to appraise the CHD effects of moderate exercise, the relevant pathogenetic pathways need to be considered. While Fig. 1 also indicates other health factors, only the pathways activated by moderate exercise are summarised in Table 1. It is however important to note that not all the pathways will be relevant to every patient and that all the pathways may not be active simultaneously, or occur in the same patient.

**Table 1:** Putative effects of moderate exercise and salient CHD pathogenetic pathways

Pathways, and pathway numbers corresponding to those in Fig. 1	References
a. 3a-53-↓ blood glucose-55-↓ hyperglycaemia	38, 39
b. 3a-53-↓ blood glucose-54-↓ PI3K:MAPK-69-↓ insulin resistance-72-↓ platelet factors-73-↓ hypercoagulability	40–47
c. 3a-53-↓ blood glucose-54-↓ PI3K:MAPK-69-↓ insulin resistance-72-↓ ROS	38, 40, 45–48
d. 3a-53-↓ blood glucose-54-28-101-↓ insulin resistance-72- ↑ vasodilation	49
e. 3b-27-↓ cortisol-47-↓ insulin resistance-70-↓ angiotensin II-89-↓ hypertension-100-↓ ROS-85-↓ COX1/2-85-↓ inflammatory state	29, 30, 38, 45, 48
f. 3b-27-↓ cortisol-47-↓ insulin resistance-70-↓ angiotensin II-89-↓ SMC proliferation	50
g. 3b-27-↓ cortisol-47-↓ insulin resistance-70-↓ angiotensin II-89-↑ IGF1-84-↓ SMC proliferation	51–54
h. 3b-27-↓ cortisol-47-↓ insulin resistance-70-↓ angiotensin II-89-↓ VCAM1/MCP1-73-↓ hypercoagulation	29
i. 3c-↓ visceral adipose tissue-↓ ectopic fat	38, 55, 56
j. 3c-19-↑ adiponectin-38-↓ TNFα/IL6-56-Liver-12-↓ LDL-33-↓ oxLDL-51-↓ hypercholesterolaemia	38, 56, 57
k. 3c-19-↑ adiponectin-39-↓ insulin resistance	58
l. 3c-19-↑ adiponectin-39-↓ SMC proliferation	55
m. 3c-21-↓ TNFα/IL6-56-Liver-12-↓ LDL-33-↓ oxLDL-51-↓ hypercholesterolaemia	5, 32, 59–62
3c-21-↓ TNFα/IL6-41-↓ P. gingivalis-43-↓ periodontitis- 64-↓ platelet factors-73-↓ hypercoagulability	5, 32, 59–62
3c-18-↓ FFA-37-↓ plasma lipids-34-Liver-12-↓ LDL-33- ↓ oxLDL-51-↓ hypercholesterolaemia	5, 32, 38, 56, 59–62

↑, up regulation/increase; ↓, down regulation/decrease; x-y-z indicates pathway connecting x to y to z. FFA, free fatty acids; IGF 1, insulinlike growth factor-1; IL6, interleukin-6; LDL, low-density lipoprotein; MAPK, mitogen-activated protein (MAP) kinase; MCP 1, monocyte chemo-attractant protein-1; NO, nitric oxide; oxLDL, oxidised LDL; P gingivalis, Porphyromonas gingivalis; PI3K, phosphatidylinositol 3-kinase; PI3K:MAPK, ratio of PI3K to MAPK; ROS, reactive oxygen species; SMC, smooth muscle cell; TNFα, tumour necrosis factor-α; VCAM 1, vascular cell adhesion molecule-1.

Fig. 1 (pathway: 3a-53-55-hyperglycaemia) shows the pathways involved in a lack of physical exercise (and decreased daily energy expenditure) and how this affects carbohydrate metabolism through changes in muscle glucose transporter (GLUT) protein content. Denervation of skeletal muscle results in rapid decreases in both muscle GLUT-4 contents and insulinstimulated glucose uptake, therefore resulting in hyperglycaemia and concomitant hyperinsulinaemia (both CHD hallmarks) in non-diabetic patients.^10^

Lack of physical exercise may also contribute to the accumulation of visceral fat, reduced lipoprotein lipase activity and reduced clearance of triglycerides, leading to increased LDL levels, decreased HDL levels, and increased LDL-to-HDL ratios, and eventually to hypercholesterolaemia.^11^ This state subsequently activates the oxidative stress/inflammation cascade. This in turn underlies insulin resistance and the evolution of micro- and macrovascular complications (Fig. 1, pathways: 3a-53-blood glucose-54-PI3K:MAPK-69-insulin resistance-72-ROS). Hyperinsulinaemia, by itself, contributes significantly to atherogenecity, leading to CHD.^12^

An increase in plasma free fatty acid (FFA) concentrations plays a key role in the pathogenesis of insulin resistance through actions that block insulin signal transduction. An increase in FFA levels results in induction of oxidative stress, low-grade systemic inflammation, and subnormal vascular reactivity, in addition to causing insulin resistance.5 As insulin resistance also results in the relative non-suppression of adipocyte hormone-sensitive lipase,^13^ there is further enhancement in lipolysis, increased FFA and insulin resistance. As insulin suppresses pro-inflammatory transcription factors, such as nuclear factor-κβ (NF-κβ), and also suppresses reactive oxygen species (ROS) generation, insulin resistance therefore also has a comprehensive pro-inflammatory effect (Fig. 1, pathways: 3c-18-FFA-37-plasma lipids-34-12- LDL-33-oxLDL-51-hypercholesterolaemia).

Fig. 1 therefore shows why an insulin-resistant state may be pro-inflammatory. The origin of the insulin resistance may be traced back to the pro-inflammatory cytokine TNF-α, which is expressed by adipose tissue.^14^ Adipose tissue has been shown to express not only TNF-α, but also other pro-inflammatory mediators, including CRP. Macrophages residing in the adipose tissue may also be a source of pro-inflammatory factors and they can also modulate the secretory activities of adipocytes15 (Fig. 1, pathway: 3c-21-TNFα/IL6).

During regular moderate exercise, IL-6 is produced by skeletal muscle fibres via a TNF-independent pathway. IL-6 stimulates the appearance in the circulation of anti-inflammatory cytokines, which inhibit the production of pro-inflammatory TNF-α.^16^ Additionally, IL-6 enhances lipid turnover, stimulating lipolysis as well as fat oxidation. Regular physical exercise therefore induces suppression of TNF-α and thereby offers protection against TNF-α-induced insulin resistance.^16^ Low-grade systemic inflammation therefore appears to be aetiologically linked to the pathogenesis of CHD,^17^ countered by moderate exercise with its anti-inflammatory effects5 (Fig. 1, pathway: 3a-53-blood glucose-54-69-insulin resistance-71).

The adipokine adiponectin is anti-inflammatory and potentially anti-atherogenic.^5^ Low adiponectin levels act as a marker for CHD and are associated with overweight subjects.^18^ Regular physical exercise (and an energy-controlled diet) reduces visceral fat mass, with a subsequent increased release of antiinflammatory adiponectin, therefore resulting in reduced risk of CHD19 (Fig. 1, pathway: 3c-19-39-insulin resistance).

Lack of physical exercise may lead to hypertension, another CHD hallmark, through increased vascular and sympathetic tone created by reduced bioavailability of nitrous oxide (NO) and activation of the renin–angiotensin system^20^, ^21^ (Fig. 1, pathway: 3a-53-blood glucose-54-60-72-vasodilation). Hypertension is directly correlated with visceral fat mass, which may be decreased by moderate exercise.^22^

The lower blood glucose levels that result from moderate exercise lead to a reduction in the phosphatidylinositol 3-kinase (PI3K) to mitogen-activated protein kinase (MAPK) ratio, which in turn decreases insulin resistance^23^ (Fig. 1, pathway: 3a-53-blood glucose-54-69-72-73-hypercoagulabilty). Increased insulin sensitivity decreases serum levels of platelet factors and thus reduces the potential for hypercoagulability.^24^,^25^

Moderate exercise also increases coronary blood flow,^26^ which increases the release of prostaglandins.^27^ This is important in heart microvasculature, in which prostaglandins are substantially involved in flow-mediated vasodilation.^27^

Moderate exercise acts on the central nervous system by decreasing serum cortisol levels.^28^ This in turn reduces insulin resistance, which decreases angiotensin II levels and results in reduced hypertension. Reactive oxygen species (ROS) and cyclooxygenase (COX) 1/2 levels reduce concomitantly, which lead to a lower inflammatory state20 (Fig. 1, pathway: insulin resistance-85-inflammatory state).

It is apparent that moderate exercise directly and indirectly affects a plethora of interconnected pathogenetic mechanisms. Each CHD hallmark and pathogenetic trait can amplify the patient’s risk of CHD, therefore necessitating an integrated, multi-faceted therapeutic approach.

In this section, the pathogenetic pathways activated by moderate exercise are described, but the effects of these pathways have not been quantified. The next interrogation was therefore whether biomarkers could quantify the CHD effect of moderate exercise. This was accomplished by using connection graphs, which link the relative effect of a health or pathogenic factor to the individual biomarkers through the pathways that are shown in Fig. 1.

## Biomarkers of coronary heart disease

The integrated model that was developed is a high-level conceptual model, from which the interconnectedness of CHD is immediately apparent (Fig. 1). The model is however complicated. Biomarkers can be used as indicators of an underlying disorder and the measurement of specific biomarkers enables prediction of the RR for CHD associated with the biomarker.^29^-^31^ The relevant biomarkers and their association with CHD risk per one standard deviation increase in said biomarker are given in Table 2. This can allow for the quantification of the effects of moderate exercise on the pathogenesis of CHD.

**Table 2:** Association between biomarkers and prediction of CHD relative risk

Biomarker (class and salient examples)	Prediction of CHD relative risk (95% CI)	Size of studies (N = number of trials, n = number of patients)	References
Lipid-related markers			
Triglycerides	0.99 (0.94–1.05)	(N = 68, n = 302 430)	63
LDL	1.25 (1.18–1.33)	(N = 15, n = 233 455)	64
HDL	0.78 (0.74–0.82)	(N = 68, n = 302 430)	63
Apo B	1.43 (1.35–1.51)	(N = 15, n = 233 455)	64
Leptin	1.04 (0.92–1.17)	(n = 1 832)	65
Inflammatory markers			
hsCRP	1.20 (1.18–1.22)	(N = 38, n = 166 596)	66
IL-6	1.25 (1.19–1.32)	(N = 25, n = 42 123)	67
TNF-α	1.17 (1.09–1.25)	(N = 7, n = 6 107)	67
GDF-15	1.40 (1.10–1.80)	(n = 1 740)	68
OPG	1.41 (1.33–1.57)	(n = 5 863)	69
Marker of oxidative stress			
MPO	1.17 (1.06–1.30)	(n = 2 861)	70
Marker of vascular function and neurohormonal activity
BNP	1.42 (1.24–1.63)	(N = 40, n = 87 474)	71
Homocysteine	1.15 (1.09–1.22)	(N = 20, n = 22 652)	72, 73
Coagulation marker			
Fibrinogen	1.15 (1.13–1.17)	(N = 40, n = 185 892)	66
Necrosis marker			
Troponins	1.15 (1.04–1.27)	(n = 3 265)	58
Renal function marker			
Urinary ACR	1.57 (1.26–1.95)	(n = 626)	74
HbA^1c^	1.42 (1.16–1.74)	(N = 2, n = 2 442)	75
IGF-1	0.76 (0.56–1.04)	(n = 3 967)	76
Adiponectin	0.97 (0.86–1.09)	(N = 14, n = 21 272)	77
Cortisol	1.10 (0.97–1.25)	(n = 2 512)	78, 79
BDNF	?	?	80–82
Insulin resistance (HOMA)	1.46 (1.26–1.69)	(N = 17, n = 51 161)	83

From: M Mathews, L Liebenberg, E Mathews. How do high glycemic load diets influence coronary heart disease? Nutr Metab 2015; 12(1): 6.^7^ n , number of participants; N, number of trials; CI, confidence interval; ACR, albumin-to-creatinine ratio; Apo B, apolipoprotein-B; BDNF, brain-derived neurotrophic factor; BNP, B-type natriuretic peptide; GDF-15, growthdifferentiation factor-15; HbA_1c_, glycated haemoglobin A_1c_; HDL, high-density lipoprotein; HOMA, homeostasis model assessment; hsCRP, high-sensitivity C-reactive protein; IGF-1, insulin-like growth factor-1; IL-6, interleukin-6; LDL, low-density lipoprotein; MPO, myeloperoxidase; OPG, osteoprotegerin; TNF-α, tumour necrosis factor-α.

**Fig. 2.**
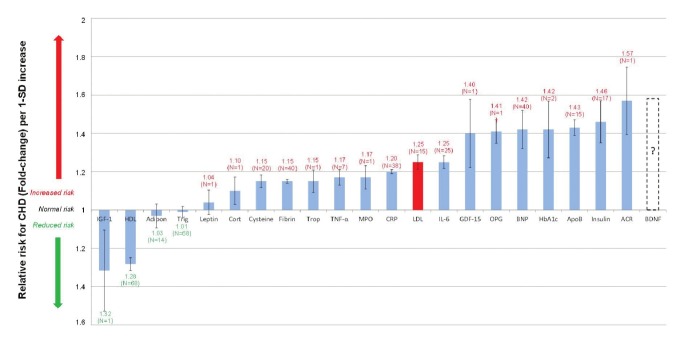
Normalised relative risks (fold-change) of salient current biomarkers or of potential serological biomarkers for CHD. (From: M Mathews, L Liebenberg, E Mathews. How do high glycemic load diets influence coronary heart disease? Nutr Metab 2015; 12(1): 6.7) Increased IGF-1 and HDL levels are associated with a moderately decreased CHD risk. (IGF-1 and HDL levels are significantly inversely correlated to relative risk for CHD.) N indicates number of trials; I, 95% confidence interval; ACR, albumin-to-creatinine ratio; Adipo, adiponectin; ApoB, apolipoprotein-B; BDNF, brain-derived neurotrophic factor; BNP, B-type natriuretic peptide; Cort, cortisol; CRP, C-reactive protein; cysteine, homocysteine; fibrin, fibrinogen; GDF-15, growth-differentiation factor-15; HbA1c, glycosylated haemoglobin A1c; HDL, high-density lipoprotein; IGF-1, insulin-like growth factor-1; IL-6, interleukin-6; LDL, low-density lipoprotein; MPO, myeloperoxidase; OPG, osteoprotegerin; TNF-α, tumour necrosis factor-α; Trigl, triglycerides; Trop, troponins.

To simplify the integrated model, serological biomarkers (which can easily be measured) are used to link the effect of exercise to the corresponding RR of CHD. Fig. 2 presents a comparison of the RR associated with an array of serological biomarkers per one standard deviation increase in the biomarker.^7^

## Effects of moderate exercise

Using the integrated model in Fig. 1, it is possible to account for the impact that moderate exercise would have on the serological biomarkers of CHD. This enables a simplification of the integrated model into a connection graph, which shows all the connections between moderate exercise and the measurable serological biomarkers.

**Fig. 3.**
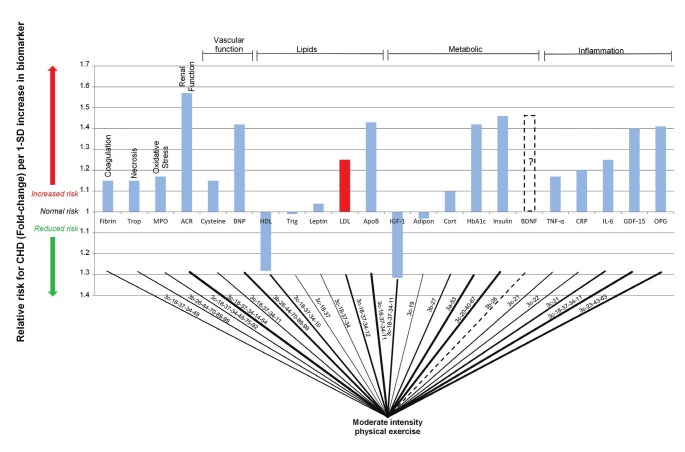
Interconnection of relative risk effects of moderate exercise and serological biomarkers for CHD. ACR, albumin-to-creatinine ratio; Adipo, adiponectin; Apo B, apolipoprotein-B; BDNF, brain-derived neurotrophic factor; BNP, B-type natriuretic peptide; Cort, cortisol; CRP, C-reactive protein; cysteine, homocysteine; fibrin, fibrinogen; GDF-15, growth-differentiation factor-15; HbA1c, glycosylated haemoglobin A1c; HDL, high-density lipoprotein; IGF-1, insulin-like growth factor-1; IL-6, interleukin-6; LDL, low-density lipoprotein; MPO, myeloperoxidase; OPG, osteoprotegerin; TNF-a, tumour necrosis factor-a; Trigl, triglycerides; Trop, troponins.

The connection graph presented in Fig. 3 does not neglect any of the underlying complexity of CHD. To more clearly determine the effect of exercise on different biomarkers in Fig. 3, the biomarkers previously shown in Fig. 2 were divided into eight classes, namely vascular function and neurohormonal activity, renal function, necrosis, coagulation, oxidative stress, lipids, and metabolic and inflammatory markers.

The pathogenetic pathways (from Fig. 1) are superimposed on the connecting lines in Fig. 3. Increasing line thickness indicates a connection with possible greater pathogenetic effect (as quantified by biomarker relative-risk prediction of CHD). For example, the risk of CHD is relatively low when considering leptin, therefore the connection line between exercise and leptin is thinner than for others (e.g. Apo B).

It is intriguing to see that moderate exercise has a connection to all the serological biomarkers. This further highlights the inverse correlation between CHD risk and moderate exercise. From the connection graph in Fig. 3, it can be noted that the potential risk reduction effect of moderate exercise may be greatly influenced by changes in inflammatory, metabolic and lipid markers, which provide a considerable increased risk for CHD.^2^-^4^

Mora and co-workers determined the mechanisms of the reduced risk of CHD associated with exercise in women.^2^ They found that a reduction in inflammatory biomarkers were the largest contributors to lowered risk. These were followed, in order, by blood pressure, lipid levels, body mass index (BMI) and haemoglobin level. In the study, the combination of different individual risk factors quantified only 35.5% of the total risk reduction due to physical exercise on CHD.^2^.

It is therefore clear that the risk factors used by Mora and co-workers, in terms of serological biomarkers, did not fully quantify the risks associated with CHD. In their study, LDL, HDL and Apo B serum levels were recorded to monitor lipid levels, but only hsCRP serum levels were used for deducing inflammatory levels.^2^ It may therefore be possible that with the addition of the other biomarkers indicated in Fig. 3, the effect of moderate exercise may be better quantified.

In Fig. 3, it is clear from the risk associated with inflammation that reduction in inflammation would prove beneficial to CHD risk. The full extent of the relationship between exercise and inflammation has not been determined but it has been proven that chronic moderate exercise has a systemic anti-inflammatory effect.^5^,^16^,^32^ It has further been shown that the anti-inflammatory effect of exercise provides the largest individual risk-reduction component of moderate exercise in women.^2^

Naturally there is a strong link to the metabolic process that is manifested in the connection to the metabolic biomarkers, specifically insulin resistance and glycated haemoglobin level.^33^,^34^ This connection may be largely mediated by the increased expenditure of energy, which produces favourable effects on CHD pathogenesis.^10^, ^23^ Moderate exercise is also related to changes in lipid factors such as increases in HDL cholesterol and decreases in LDL cholesterol and Apo B levels.^33^,^34^

## Discussion

It is clear that there are a wide variety of effects of exercise on the pathogenesis of CHD, which can be described by the changes in biomarkers. However, from the connection graph in Fig. 3, it is not immediately clear what the overall effect of moderate exercise is on CHD. This effect has been quantified in the RR reduction for CHD, which is observed in those who engage in moderate exercise.

Moderate-intensity physical exercise of 1 100 kcal/week is associated with an average RR of 0.75 (0.71–0.79), based on a large meta-analysis.^35^ The RR of 0.75 would correlate to a RR reduction of 1.33-fold using the method previously described in the Methods section.

The data from Fig. 3 show that inflammation and metabolic dysregulation may be key aspects in the pathogenesis of CHD.^5^,^10^,^16^,^23^,^32^-^34^ These aspects decrease during exercise and may therefore play a part in the 1.33-fold decreased risk for CHD.

Based on the evidence, it is believed that the CHD benefit associated with exercise is substantial and should garner a similar level of public interest as do other risk factors such as smoking, high cholesterol levels and treatments such as statin therapy. However, while exercise is frequently advised for healthy living,36 it is unfortunate that only 48.9% of Americans meet the physical activity guidelines. It follows from this that 51.1% of Americans do not meet the minimum physical activity guidelines, which results in 162.8 million Americans at a greater risk of CHD due to physical inactivity.^37^

The individual studies selected unfortunately represent only the risk associated with the cohort studied and cannot accurately be extrapolated to other populations without further research.

## Conclusion

Although it is well known that moderate exercise is associated with a lower risk of CHD, all the positive effects on CHD pathogenesis were not available in a detailed integrated model. Such a model would help provide further insight. A high-level conceptual model was therefore developed, which links moderate exercise with the pathogenesis, hallmarks and biomarkers of CHD.

The novel connection graph developed from this model shows, at a glance, the positive effect of moderate exercise on certain important aspects of the pathogenesis of CHD. It helps to graphically explain why moderate exercise is associated with lower CHD risk. From this it is apparent that exercise has a wideranging impact on the pathogenesis of CHD, with these effects notable in changes in CHD biomarkers.

The integrated high-level CHD model and simplified connection graph provide a summary of evidence for a causal relationship between CHD risk and moderate exercise. We acknowledge the fact that the integrated view is relevant to other lifestyle issues and for full comprehension will have to be replicated in other articles describing these factors.
